# Supplementary material for: Mapping the evolution of fertility support policies in China: A content and instrumental analysis
Source: PLoS One. 2025 Oct 9;20(10):e0332137. doi: 10.1371/journal.pone.0332137 (PMC12510515; doi:10.1371/journal.pone.0332137)
Supplement: S1 Appendix — (ZIP) [file pone.0332137.s001.zip › S1 Appendix. 226 original policy documents/133-乡镇卫生院中医药服务管理基本规范(FBM-CLI-4-50790).doc]

乡镇卫生院中医药服务管理基本规范

**发布部门：** [卫生部(已撤销)](https://www.pkulaw.com/law/chl?Aggs.IssueDepartment=60222) [国家中医药管理局](https://www.pkulaw.com/law/chl?Aggs.IssueDepartment=7060306) [机构沿革](javascript:void(0))

**发布日期：**2003.11.25

**实施日期：**2003.11.25

**时效性：** [现行有效](https://www.pkulaw.com/law/chl?Aggs.TimelinessDic=01)

**效力级别：** [部门规范性文件](https://www.pkulaw.com/law/chl?Aggs.EffectivenessDic=XE0302)

**法规类别：** [卫生机构与人员](https://www.pkulaw.com/law/chl?Aggs.Category=09602)

乡镇卫生院中医药服务管理基本规范
（卫生部、国家中医药管理局　2003年11月25日印发）

　　一、总则
　　(一)为加强乡镇卫生院中医药服务规范化管理，发挥中医药在农村卫生工作中的优势与作用，根据《[中华人民共和国中医药条例](https://www.pkulaw.com/chl/f09160506034409fbdfb.html)》，制定本规范。
　　(二)本规范适用于依法设立的乡镇卫生院。乡镇卫生院民族医药服务管理，以及其他基层乡镇卫生机构中医药服务管理，参照执行。
　　(三)县级以上地方人民政府负责中医药管理的部门负责对本行政区域内乡镇卫生院中医药服务进行监督管理，并安排专人负责。
　　省、自治区、直辖市人民政府负责中医药管理的部门应当结合行政区域内实际，制定切实发挥中医药在农村优势与作用的具体政策措施和乡镇卫生院中医科基本设施配置标准，确定乡镇卫生院中医药业务工作的具体指标值。省、自治区、直辖市卫生行政部门应当把中医药服务项目纳入新型农村合作医疗的支付范围。
　　二、中医科建设
　　(四)乡镇卫生院应当将提供中医药服务作为其业务工作的重要内容，设置中医科，开设中药房，有相应的专用医疗用房。房屋面积、环境等达到省级中医药管理部门规定的要求和标准。
　　(五)有条件的乡镇卫生院可根据本地区疾病谱、中医药专业技术条件等情况开设相应的特色专科(专病)，设置中药炮制室、煎药室。
　　三、中医药人员配备和人才培养
　　(六)乡镇卫生院应当建立稳定的中医药专业技术人员队伍。
　　中医药专业技术人员，应当依照有关法律法规取得执业资格，并经注册取得执业证书后，方可从事中医药服务活动。
　　中医执业助理医师和中医执业医师应占执业助理医师和执业医师总数的一定比例，具体比例由省、自治区、直辖市卫生行政部门制定。
　　(七)应当开展多种形式的岗位培训、在职教育和学术交流，提高中医药专业技术人员学历层次和实际工作能力。
　　中医药专业技术人员每五年参加进修学习的时间为3～6个月以上。
　　鼓励农村临床医疗服务人员兼学中医，并应用中医药诊疗技术为农民服务；加强农村临床医疗服务人员的中医药知识与技能的培训。
　　四、中医药服务基本内容
　　(八)医疗服务
　　1．提供基本的中医医疗服务，在门诊、病房、出诊、家庭病床等工作中运用中医理论辨证论治处理常见病、多发病、慢性病；
　　2．根据“简、便、验、廉”的原则，运用包括中药、针灸、推拿、火罐、敷贴、刮痧、熏洗、穴位注射、热熨等在内的5种以上中医药适宜技术；
　　3．运用中医药方法结合现代理疗手段，开展中医康复医疗服务；
　　4．提供中成药和中药饮片品种数量应当满足开展中医药服务需要。中成药品种应当在80种以上，中药饮片应当在250种以上。经济欠发达地区，可适当调整。
　　(九)预防保健
　　1．充分发挥中医药特色和优势，积极参与辖区内传染病的预防工作；
　　2．开展2种以上常见病、多发病、慢性病中医药防治一体化的服务，运用中医理论和技术，参与健康指导和行为干预；
　　3．制定有中医药内容的适合辖区内老年人、妇女、儿童等重点人群以及亚健康人群的保健方案，并组织实施。有条件的，应开展具有中医特色的养生保健；
　　4．运用中医药知识开展优生优育、生殖保健和孕产妇保健的咨询及指导；
　　5．运用多种形式，宣传中医药防病、保健知识，能够提供有中医药内容的健康教育。
　　(十)提供中医药服务应当严格遵守国家有关中医诊断治疗原则、医疗技术标准和技术操作规范。
　　五、加强村卫生室的中医药业务管理和指导
　　(十一)乡镇卫生院应开展对村卫生室的中医药技术指导、业务管理和对乡村医生的培训，使每个村至少有一名中医或能中会西的乡村医生；并指导乡村中医药技术人员积极利用当地中医药资源，自种、自采、自用中草药。
　　开展乡村卫生服务管理一体化的地区，乡镇卫生院应当承担村卫生室中成药和中药饮片统一代购工作，保证中药质量和用药安全。
　　(十二)县级以上地方人民政府负责中医药管理的部门应当将村卫生室的中医药业务工作列入乡镇卫生院综合目标考核内容。

© 北大法宝：（[www.pkulaw.com](https://www.pkulaw.com/)) 专业提供法律信息、法学知识和法律软件领域各类解决方案。北大法宝为您提供丰富的参考资料，正式引用法规条文时请与标准文本核对。
欢迎查看所有[产品和服务](https://www.pkulaw.com/helps/2.html)。法宝快讯：[如何快速找到您需要的检索结果？](https://www.pkulaw.com/helps/70.html)[法宝](https://www.pkulaw.com/helps/69.html)V6有何新特色？

扫描二维码阅读原文

**原文链接：**<https://www.pkulaw.com/chl/bcee205ebf9473d0bdfb.html>
